# Development and validation of a questionnaire assessing pharmacists' knowledge and practice towards antimicrobial stewardship in oncology care

**DOI:** 10.1371/journal.pone.0321551

**Published:** 2025-05-23

**Authors:** Ali Haider Mohammed, Bassam Abdul Rasool Hassan, Lim Jie Sern, Lim Jing Xuan, Leong Jia Yun, Lim Jing En, Dinesh Sangarran Ramachandram, Angelina Lim, Juman Abdulelah Dujaili, Ali Qais Blebil

**Affiliations:** 1 School of Pharmacy, Monash University Malaysia, Jalan Lagoon Selatan, Bandar Sunway, Subang Jaya, Selangor, Malaysia; 2 Department of Pharmacy, Al Rafidain University College, Baghdad, Iraq; 3 Faculty of Pharmacy and Pharmaceutical Sciences, Monash University, Parkville, Victoria, Australia; 4 Murdoch Children's Research Institute, Royal Children's Hospital, Parkville, Victoria, Australia; 5 Swansea University Medical School, Swansea University, Swansea, United Kingdom; Lahore Medical and Dental College, PAKISTAN

## Abstract

**Background:**

Cancer patients who receive immunosuppressive therapy are vulnerable to infections due to their compromised immune systems. Therefore, research in promoting the prudent use of antibiotics in this population is essential to optimise patient outcomes and reduce resistance development. The purpose of this study is to develop and validate the Knowledge and Practice of Antimicrobial Stewardship in the Oncology Care Questionnaire (KP-AMS-OC-Q).

**Method:**

This research is performed in 2 phases. Phase I includes the questionnaire development, item generation, content validity and pilot testing. Phase II encompasses the dissemination of questionnaires to hospital pharmacists and the psychometric evaluation of the validation and reliability of KP-AMS-OC-Q. Specifically, IRT is used to evaluate the knowledge domains, EFA for the practice domains, and Cronbach alpha to measure reliability.

**Result:**

The finalised version of the questionnaire consisted of 112 items, including 7 social demographics, 49 knowledge, and 56 practice items. IRT conducted has revealed an acceptable difficulty parameter (-3 to +3) in the knowledge domain. Furthermore, EFA has shown a strong internal association between the items and factors, with each item reaching the minimum acceptable factor loading value (>0.3). Besides this, the internal consistency of this questionnaire is favourable, indicated by the Cronbach alpha coefficient of 0.899.

**Conclusion:**

The results of this study have validated that KP-AMS-OC-Q possesses exceptional psychometric qualities, making it appropriate to measure pharmacists’ knowledge and practice towards antimicrobial stewardship (AMS) in oncology care.

## Introduction

In the ever-changing scenery of healthcare, antimicrobial resistance (AMR) has become a public health issue that requires immediate action from all parties in combating the danger that it can bring to the public. AMR develops as bacteria evolve due to antimicrobial exposure. This evolutionary adaptation is hastened by the excessive or inappropriate use of antimicrobial drugs. Of particular interest, the rising trend of antimicrobial usage can also be seen clearly in Asian countries, even in Malaysia. As of 2021, a research in Malaysia has shown that the prevalence of antimicrobial use was 49% during the patients’ stay in the hospital [1] Increased antimicrobial use is correlated with the emergence of AMR as can be shown in a study in 2018. Resistance to specific antimicrobial drugs can increase when the consumption of those drugs passes a specific threshold [2]. This is of great concern as it can be seen that there is a lack of improvement shown within these years in the crucial area to fight against AMR. In fact, the antimicrobial prescription rate in the oncology setting was one of the highest with a proportion of 57.1% [1] which leads back to the topic regarding the implementation and persistent development of antimicrobial stewardship (AMS) in the oncology setting.

In the context of rising AMR in Asian countries, the focus on oncology care within the broader discussion of AMS becomes even more critical. Oncology patients who undergo treatment like chemotherapy and radiation therapy have weakened immune systems making them highly susceptible to infections. Therefore, the wise use of antimicrobials in this setting is crucial for treating infection effectively and averting the emergence of resistance. This is significant as resistance can harm patient outcomes and limit treatment options.

Research conducted by the Asian Network for Surveillance of Resistant Pathogens (ANSORP) has demonstrated a high prevalence of erythromycin resistance across the region, with rates reaching 72.7%. In particular, China exhibited a resistance rate of 96.4%, followed by Taiwan at 84.9%, and Vietnam at 80.7% [3]. These infections caused by multidrug-resistant organisms (MDRO) are easily spreadable and can lead to treatment failure and subsequently, increased mortality and morbidity. Prolonged neutropenia further complicates the matter, as it predisposes cancer patients to infections, necessitating extended and repeated courses of broad-spectrum antibiotics, which is a leading cause of MDRO development [4]. Besides, hospitalised cancer patients with multiple and recent antimicrobial exposures may have higher than necessary drug exposure values that can lead to drug toxicity instead.^5^ Due to the high interpatient PK variability [5] and minimum inhibitory concentration (MIC) distribution of pathogens that further complicates antimicrobial dosing in oncology patients, tailored guidelines should be formulated in aid of treatment for this population to balance the risk and benefits of antimicrobial activity. Furthermore, failure in antibiotic treatment is correlated with increased risk of sepsis, mortality rate and healthcare cost. This is further complicated by the fact that chemotherapy may encourage microbial evolution and increased appearance of mutant bacteria in patients who have developed antibiotic resistance [6]. A study by Raheem et. al. in 2020 [7] highlights the critical role of AMS adherence in managing AMR, showing that pharmacist-led AMS initiatives are particularly effective in reducing resistance rates, even in outpatient settings. This study underscores the importance of robust AMS practices in controlling the spread of MDROs and improving patient outcomes, further emphasizing the need for similar targeted AMS strategies in oncology care

Throughout the years, there has been limited research in evaluating pharmacist’s knowledge and practices in AMS, especially in oncology care. For instance, a study done in 2021 by Mubarak et. al [8]. have discussed the implementation of AMS practices within hospital settings, and underscored the important contributions pharmacists make in optimizing antimicrobial use and preventing resistance. However, although these studies have explored the broader role of pharmacists in AMS implementation in hospital settings, they often fail to address the unique needs of oncology patients. This reflects a significant gap of how healthcare professionals deal with the complexities of antimicrobial management in oncology patients, and the importance of pharmacists’ involvement in determining the fine balance between the judicious and effective use of antimicrobials in managing patients’ infections and preserving oncological treatment outcomes. As drug experts, pharmacists would need to deal with the specific challenges of antimicrobial use in oncology settings which include issues such as drug-drug interactions (DDIs) and the emergence of MDRO which requires tailored and individualised approaches. On that account, further research is also necessary to explore pharmacists’ knowledge and practice regarding AMS in oncology to reduce AMR through timely clinical intervention and improve patient outcomes in the vulnerable population.

Up to date, there are no existing assessment tools that are designed particularly to evaluate the knowledge and practice of pharmacists towards AMS in oncology care. Thus, the aim of this study is to develop and evaluate a comprehensive tool evaluating pharmacists’ knowledge and practices towards AMS in oncology care, providing a better understanding of pharmacists’ contributions.

## Methodology

### Study design

A cross-sectional survey was selected as the instrument of choice to develop and validate the questionnaire assessing pharmacists’ knowledge and practice towards AMS in oncology care. This research design was utilised to effectively examine the knowledge and practice of health personnels and allowing validation and reliability studies to be done [9]. The Knowledge and Practice of Antimicrobial Stewardship in Oncology Care Questionnaire (KP-AMS-OC-Q) consisted of two distinct phases. The first phase includes the generation of items through a rigorous examination of relevant literature, which is then reviewed by experts in a single round of the Delphi technique. The second phase of KP-AMS-OC-Q involves the evaluation of the psychometric properties of this questionnaire, in particular its factorability and reliability.

### Study setting

This study is conducted in Malaysia, a nation in Southeast Asia where there is a high cancer incidence rate, with an estimation of 155,507 cancer cases occurring in the past 5 years [10]. Besides, there is an increased AMR rate of penicillin, erythromycin and gentamicin towards Staphylococcus aureus when compared to 2022 according to the National Antibiotic Resistance Surveillance Report 2023 in Malaysia. Additionally, reports have indicated a rise in vancomycin resistance towards Enterococcus faecalis [11].

Throughout the years, infections have grown to be the second leading cause of death among cancer patients [12]. This is further supported with research done by Zheng et al., demonstrating that the mortality rate of a cancer patient from deadly infection is three times higher than the normal population [13]. Despite the severity of this issue, few studies have been conducted in Malaysia to evaluate the knowledge and practice of AMS among pharmacists [14,15]. However, they are generally concentrated on hospital settings or comparisons among different hospitals without a specific focus on oncology care. Therefore, Malaysia is a suitable study location to conduct this research.

### Study population

The study population comprised adult Malaysian individuals who are hospital pharmacists employed in the hospital sector. The exclusion criteria were carefully selected to omit those who are not registered as pharmacists, do not work as hospital pharmacists, are illiterate, or are non-Malaysian citizens. These criteria aimed to specifically assess the knowledge and practices of pharmacists regarding AMS in oncology settings found in hospitals. This approach allows the general applicability of study findings for identifying the gaps in pharmacists’ knowledge and practice in this sector. It also opens avenues for further research such as development of targeted interventions and strategies to enhance the skills and knowledge of pharmacists in Malaysia.

### Study instrument

The study utilised a multi-phase design to ensure the developed questionnaire is valid and reliable for measuring the intended responses from the target participants. This included Phase I: Questionnaire Development and Phase II: Psychometric Evaluation of the KP-AMS-OC-Q.

Phase l: Questionnaire development

**Fig 1:**
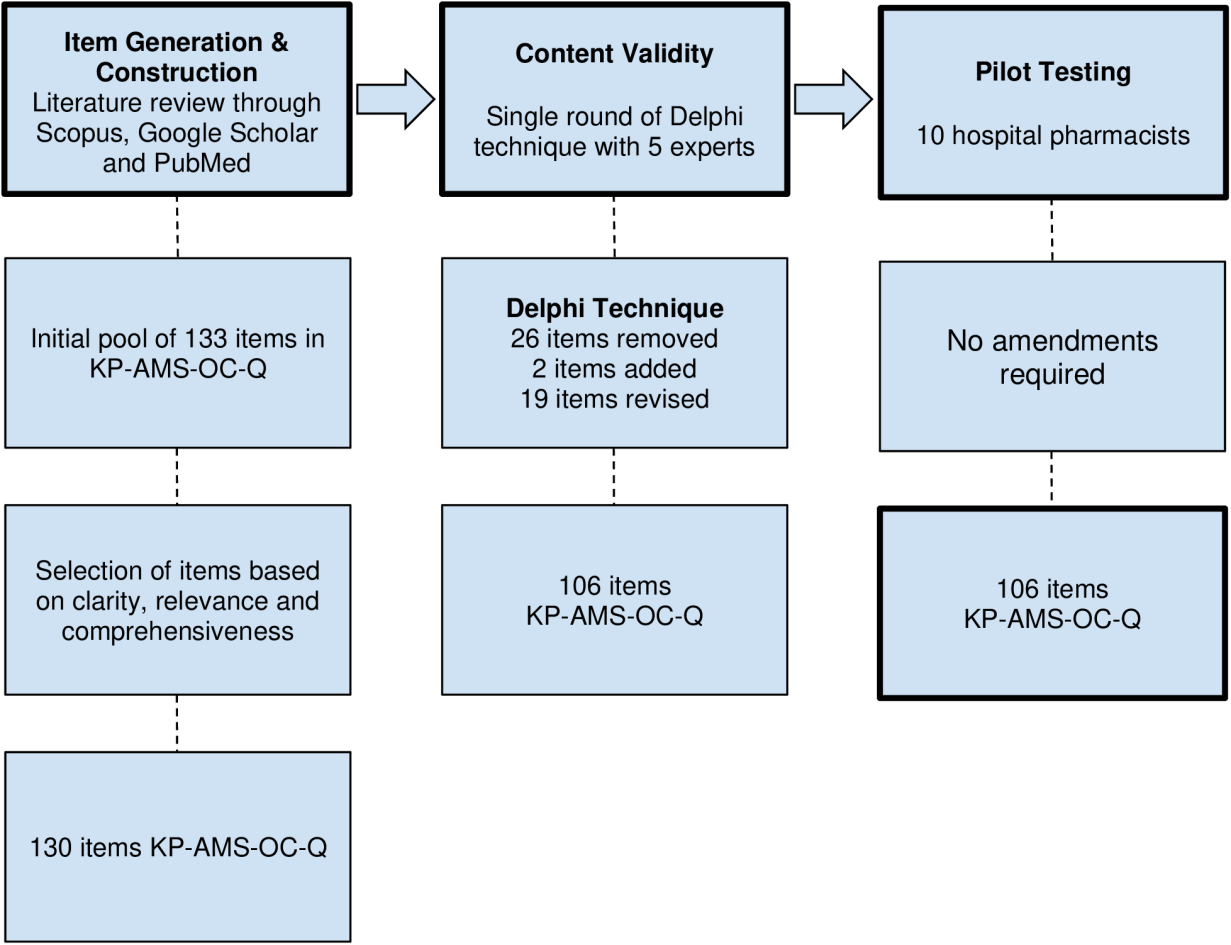
Summary of stages in Phase I.

The development of KP-AMS-OC-Q was executed in three stages which includes item generation and construction, content validity and pilot testing as shown in Fig 1.

Stage 1: Item generation and construction

Extensive literature reviews were guided using reliable research databases such as Google Scholar, PubMed, and Scopus. Search terms including “antimicrobial resistance,” “antimicrobial stewardship”, “knowledge and practice,” “oncology care/cancer patients”, and “pharmacist” were utilized to aid in the research process [4,6,16–22]. The literature review helped in the understanding and formulation of relevant domains and questions which are useful to contemplate about pharmacists’ knowledge and practice in AMS in the oncology setting.

The knowledge section of the questionnaire were divided into 2 parts, with the first part focusing on pharmacists’ general understanding in antimicrobial usage such as general knowledge about AMS, goals of AMS program, pharmacists’ roles in AMS, trends and risk factors of AMR, and clinical guidelines and protocols regarding antimicrobial use. Diving deeper into the oncology setting, the questionnaire is set out to review the pharmacists’ knowledge in infections and their management in oncology patients, PK-PD of antimicrobials in cancer patients as well as the association between chemotherapy and AMR.

Besides that, the practice section of the questionnaire focused on pharmacists’ practice in assessment and decision making of antimicrobial use, implementation of AMS strategies within the clinical field along with interdisciplinary collaboration with other healthcare professionals. Questions related to pharmacists’ practice in provision of patient education and counselling, education and training in relation to AMS in oncology care and the management of DDIs between chemotherapy and antimicrobials were also included in the survey.

Based on ideas obtained via comprehensive literature reviews, a total of 133 questions were generated for the initial questionnaire. Questions were then refined to 130 questions for both domains according to the relevance, comprehensiveness, clarity and understandability towards the target population who are hospital pharmacists.

Stage 2: Content validity

Delphi technique was used to evaluate the KP-AMS-OC-Q content validity in order to determine how relevant the questionnaire was to the research topic. A single round of the Delphi technique was conducted in this study due to time and resource constraints. Given the study timeline and the availability of experts, conducting additional rounds would have posed logistical challenges without necessarily yielding substantial improvements in content validity. Delphi technique is a method which involves collecting opinions and feedback from a panel of experts who are knowledgeable about this topic for the research team to make more informed predictions and decisions. In this process, five experts with extensive clinical background were chosen but remained anonymous to each other. The diversity in background ensured that a variety of perspectives were being considered, hence reducing the risk of bias. Majority of the expert panel was made up of oncologists, hospital pharmacists who are working mainly in the oncology unit and academic lecturers with a range of clinical specializations and backgrounds. They were requested to rate the relevance of the item to the topic using a 4-point Likert scale, with a score of 1 (not relevant) to 4 (extremely relevant). Items with an I-CVI value of less than 0.78 were removed, while those with a value of at least 0.78 were retained [23]. Besides this, the expert panels’ feedback has been taken into account while the items are being revised and modified.

During the Delphi approach, the expert panel reviewed the 130 items in the KAP-AMS-OC-Q. After collecting responses from the panel, it was identified that a total of 26 items were irrelevant and were subsequently removed from the questionnaire. Within the removed questions, 1 question in particular was split into 2 individual items, which were added in response to experts’ suggestions. Additionally, 19 items were modified based on the expert input, while 1 item was relocated from one domain to another. As a result, the finalized version of KAP-AMS-OC-Q consists of 106 items, with 50 items in the knowledge domain and 56 items in the practice domain, as demonstrated in Table 1.

**Table 1:** Changes in KP-AMS-OC-Q based on Delphi method assessment.

Original Item	Changes Made	Status After Round	Revised/Final Item
Pharmacists are facing challenges in carrying out antimicrobial stewardship (AMS) in Malaysia due to the lack of resources in terms of training, manpower and facilities as well as attitudinal challenges.	–	Removed	–
Establishment of an infectious diseases clinical pharmacist program is unable to improve antibiotic prescribing practices even in resource-limited settings.	–	Removed	–
Antimicrobial cost is the primary aim of AMS therefore it is used to justify antimicrobial stewardship programs.	–	Removed	–
Antimicrobial drugs are infinite resources which affect the growth, and ecology of invading pathogens and commensal microorganisms.	–	Removed	–
More than 50% of pharmacists viewed the AMS program as vital in improving patient care.	–	Removed	–
Pharmacy-led audit and feedback has no role in monitoring the appropriateness of antimicrobial prescriptions and compliance to AMS recommendations.	–	Removed	–
Pharmacists contribute to AMS by failing to choose the most suitable antibiotics and disregarding patient-specific characteristics in the process.	–	Removed	–
Timely microbiological culture review obtained from cancer patients by pharmacists is not important in the determination of therapy appropriateness.	–	Removed	–
AMS strategy frameworks should only focus on the appropriate use of antimicrobials.	–	Removed	–
Pharmacists play a minimal role in the implementation of antimicrobial stewardship strategies	–	Removed	–
It has been noted that the consumption of antibiotics were higher when the antibiotic advisor was a pharmacist and the pharmaceutical team reviewed the prescriptions	–	Removed	–
In the future years, the deaths due to cancer will exceed the deaths due to AMR.	–	Removed	–
By 2050, approximately 10 million people worldwide will die every year due to antimicrobial resistance.	–	Removed	–
E.coli only shows resistance towards cephalosporin class antibiotics.	–	Removed	–
Electronic alerts and educational materials do not help in promoting compliance with local and national guidelines.	–	Removed	–
More than 50% of cancer related deaths are caused by infection.	–	Removed	–
More than 50% of patients with haematologic malignancies remain afebrile during neutropenia associated with chemotherapy.	–	Removed	–
Guidelines from the Infectious Diseases Society of America (IDSA) listed cephalosporins as the only therapy for hospitalised cancer patients with neutropenic fever.	–	Removed	–
Up to date, there has not been any studies regarding optimised dosing regimens for antimicrobial use in cancer patients.	–	Removed	–
Efavirenz is able to induce CYP2C9 enzymes, resulting in increased clearance of vinca alkaloids (e.g., vincristine) and decreased serum concentrations of vinca alkaloids.	–	Removed	–
I dispense antimicrobials for a duration longer than prescribed by the health practitioner as per the patient’s request.	–	Removed	–
I do not bother to educate other healthcare professionals in regards to AMR and AMS.	–	Removed	–
I dispense patients’ antimicrobial prescription without understanding their knowledge regarding the medication.	–	Removed	–
During outpatient antibiotic prescribing, I include a combination of evidence-based practices with effective patient communication to address the patient's psycho-social factors such as the fear of medication complications.	–	Removed	–
I only counsel my patients about the dose and frequency of their antimicrobials.	–	Removed	–
I recommend my patients to stop taking their antibiotics when their symptoms have improved.	–	Removed	–
–	I always try to utilise best communication practices when counselling on antibiotics to ensure patient's take their medications properly.	Added	P32
–	I always address any medication-related queries or concerns during my counselling of antibiotics to enhance adherence.	Added	P33
AMS is associated with increased mortality rate, morbidity rate and healthcare cost.	Rewording	Revised	K1: Antimicrobial stewardship (AMS) is associated with increased mortality rate, morbidity rate and healthcare cost.
Antimicrobial Stewardship Program (ASP) in oncology requires interdisciplinary collaboration between pharmacists, oncologists, haematologists and infectious disease physicians.	Rewording	Revised	K2: AMS in oncology only involves pharmacists.
Antimicrobial stewardship programs (ASP) involve a number of core elements and action to promote the appropriate use of antimicrobials while preserving them for the future.	Rewording	Revised	K3: AMS helps to promote the appropriate use of antimicrobials and preserve them for the future.
ASP should only be implemented in primary hospitals prescribing antibiotics.	Rewording	Revised	K4: AMS should only be implemented in tertiary hospitals.
The AMS program was only implemented to reduce abuse and misuse of antimicrobials.	Rewording	Revised	K6: AMS was only implemented to reduce abuse and misuse of antimicrobials.
ASPs are able to reduce antimicrobial therapy duration only.	Rewording	Revised	K7: AMS are able to reduce antimicrobial therapy duration only.
Decreasing the emergence, selection and spread of AMR by optimising antimicrobial use is the primary goal of AMS.	Rewording	Revised	K8: Decreasing the emergence, selection and spread of antimicrobial resistance (AMR) by optimising antimicrobial use is the primary goal of AMS.
More than 50% of ambulatory patients were prescribed broad-spectrum antibiotics unnecessarily.	Rewording	Revised	K12: Majority of ambulatory patients were prescribed broad-spectrum antibiotics unnecessarily.
Cancer patients are at higher likelihood of developing antibiotic resistance and producing selection pressure due high antimicrobial usage for various indications (e.g., antimicrobial prophylaxis, targeted therapy).	Rewording	Revised	K18: Cancer patients are at higher likelihood of developing antibiotic resistance through selection pressure due to needing multi-drug regimens to treat ongoing/multiple infections.
Clinical guidelines can be developed to focus on the approach towards febrile neutropenia, antifungal prophylaxis in neutropenia as well as CMV treatment and prophylaxis.	Rewording	Revised	K21: Clinical guidelines are developed to focus on the approach towards febrile neutropenia, antifungal prophylaxis in neutropenia as well as CMV treatment and prophylaxis.
It is advised that new clinical guidelines be led by both an infectious disease physician (ID) and a pharmacist with ID training.	Rewording	Revised	K23: Guidelines for clinical practices are recommended to be developed collaboratively by an infectious disease physician (ID) and a pharmacist with ID training to ensure comprehensive expertise.
Prolonged episodes of neutropenia and repeated courses of immunosuppressive agents does not affect the vulnerability of cancer patients towards infections.	Rewording	Revised	K25: Prolonged episodes of neutropenia and repeated courses of immunosuppressive agents do not diminish the susceptibility of cancer patients to infections.
Higher antimicrobial exposure is required in patients receiving oncology care due to profound neutropenia.	Rewording	Revised	K34: Cancer patients are more prone to opportunistic infections compared to non-cancer patients.
I dispense all repeated antimicrobial prescriptions without confirming its necessity.	Rewording	Revised	P4: I dispense all repeat antimicrobial prescriptions without confirming its necessity.
I delve into international guidelines and primary literature to support my recommendation to consultants.	Rewording	Revised	P6: When recommending AMS interventions to my consultants, I always provide evidence that comes from international guidelines or primary literature.
I monitor and optimise antimicrobial prescriptions.	Rewording	Revised	P8: I review every prescription for appropriate antimicrobial use and intervene when necessary.
I do not take a patient's antimicrobial allergies and adverse reactions into consideration.	Rewording	Revised	P10: I always take a patient's antimicrobial allergies and adverse reactions into consideration.
I find it challenging to develop an efficacious ASP in community and/or hospital pharmacists due to lack of infectious disease (ID) pharmacists.	Rewording	Revised	P25: I find it challenging to develop efficacious AMS programmes in community and/or hospital pharmacists due to lack of infectious disease (ID) pharmacists.
I provide regular feedback to other healthcare professionals (e.g., oncologist, physicians) in the administration, dispensing and monitoring of antibiotics in oncology patients.	Rewording	Revised	P26: I provide regular feedback to other healthcare professionals (e.g., oncologist, physicians) regarding administration, dispensing and monitoring of antibiotics in oncology patients.

Stage 3: Pilot testing

A pilot test among 10 hospital pharmacists who fit the inclusion criteria was recruited to ascertain the clarity, understandability, and feasibility of the questionnaire. A small number of hospital pharmacists are selected here because the primary goal is to identify any potential issues regarding the clarity and feasibility of the questionnaire rather than conducting a full-scale psychometric testing. Participants are requested to go through each item on the KP-AMS-OC-Q (106 items), make remarks on the clarity of the statement provided, and state any ambiguity within it. After feedback was collected and analysed, it revealed that no corrections were needed and that the questionnaire could be completed within a time frame of approximately 15 minutes. Hence, the finalised draft of the questionnaire is unaltered and is prepared for data collection among the hospital pharmacists in Malaysia as shown in Table 2.

**Table 2:** KP questionnaire towards AMS in oncology care among hospital pharmacists in Malaysia.

Domain	No. of Items	Measurements	Responses
Social demographics	7	Age, Gender, Education, Place of education, Pharmacist qualification, Job experience, Job scope	Open-ended, close-ended
Knowledge	50	General knowledge regarding AMS, goals of AMS programs, roles of pharmacists in AMS, trends in AMR, risk factors of AMR occurrence, clinical guidelines and protocols, infections in cancer patients, management of infections in cancer patients, PK-PD of antimicrobials in cancer patients, association between chemotherapy and AMR, association between immunotherapy and AMR, DDIs between antimicrobials and chemotherapy	Yes/No/Unsure
Practice	56	Assessment and decision making, implementation of AMS strategies, interdisciplinary collaboration, patient education and counselling, pharmacists’ education and training, management of DDIs between chemotherapy and antimicrobial	Never/Rarely/Sometimes/Often/Always

Phase ll: Psychometric evaluation of the KP-AMS-OC-Q

**Fig 2:**
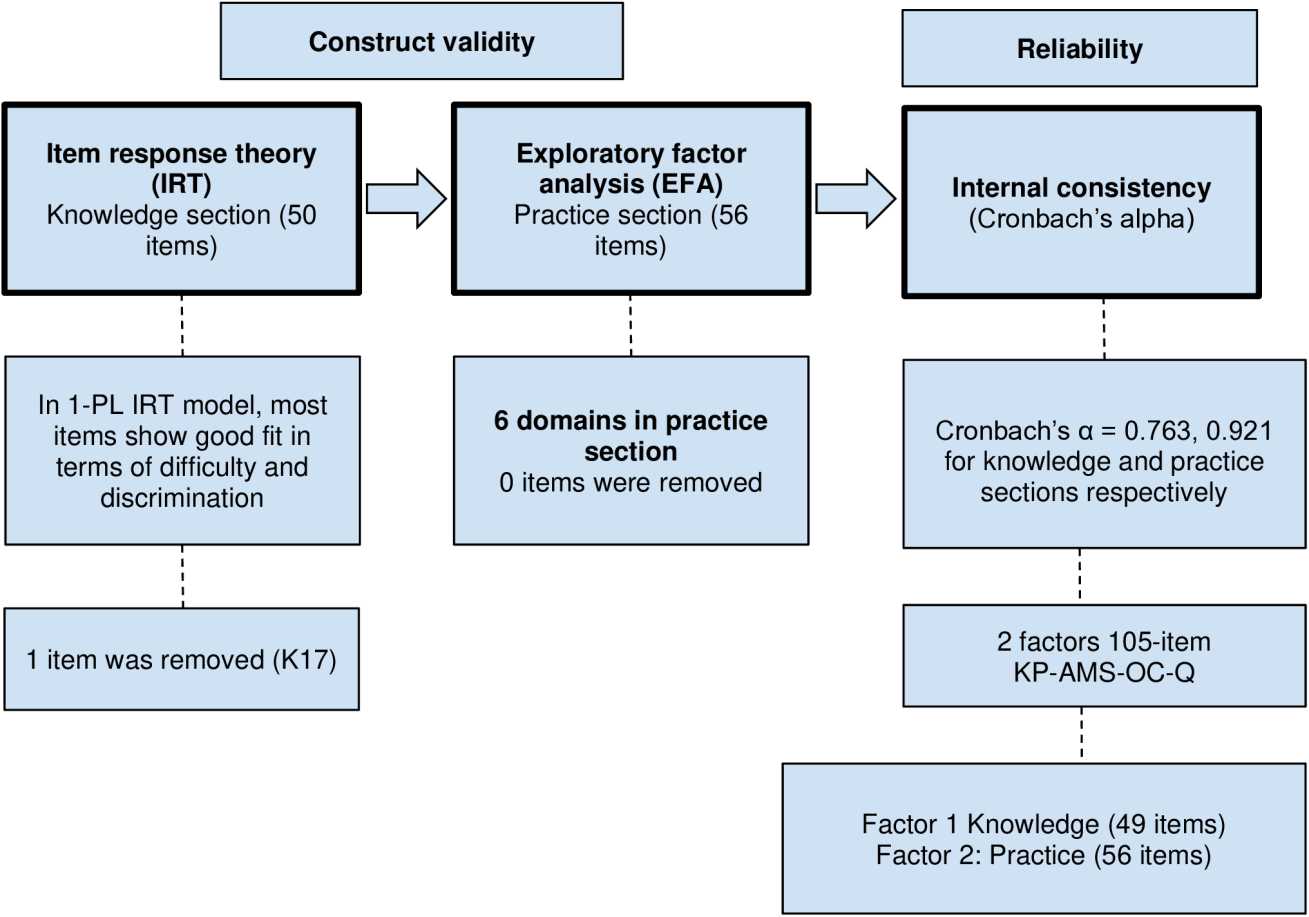
Construct validity and reliability of the questionnaire.

Phase II consists of the dissemination of the 106-item KP-AMS-OC-Q to hospital pharmacists employed in government and private hospitals in Malaysia. Using psychometric methods, this phase aimed to evaluate the questionnaire’s construct validity and reliability. The sequential steps of the process involved were represented in Fig 2.

### Study sampling

Previous research has shown that a minimum sample size of 30 participants from the population of interest is typically recommended for a pilot study aiming for conducting a preliminary survey [24]. This sample size is sufficient to offer statistical significance for testing the psychometric properties of the questionnaire.

### Data collection procedure

Prior to data collection, the research team has obtained ethical approval. The questionnaire was then created in a Google Document and then transferred to SurveyMonkey^®^ to aid in the disseminating process by sharing the survey link. The recruitment process in disseminating the questionnaire was done via online platforms such as WhatsApp, Facebook, Instagram, Emails and LinkedIn. Various social media platforms were used to ensure that participants are of different social demographics which include age, gender, qualification in pharmacy, education setting, duration of job experience and job scope in the hospital.

For eligibility of this study, participants were required to meet the inclusion criteria that were stated above. The process of recruitment involved sending the survey link and providing basic information regarding the study to invite interested participants to click on the survey link, which brings them to then questionnaire collection portal in SurveyMonkey.

Data collection took place from February to May 2024. Throughout this period, a total of 128 emails and reminders were sent to the heads of pharmacy departments in respective hospitals across Malaysia with requests to aid us in distributing the questionnaire in their pharmacy departments. The questionnaire was also posted in Facebook groups such as the Malaysia Pharmacist group as well as on LinkedIn to recruit pharmacists who are active in the platform. However, the response rate was 54.74%, as only 52 participants out of 95 completed the entire questionnaire at the end of the data collection phase.

In the beginning of the survey, the purpose of the survey and a link to the full version of the explanatory statement were provided before the participants decide whether or not to participate in the study. The inclusion criterias, a brief introduction of the study and the privacy and confidentiality of participants’ personal information and responses were explained thoroughly. Participants were informed that the collected information would be handled professionally and confidentially and that their personal information will not be linked to the data collected. They were requested to provide their consent before assessing the questionnaire. Contact information of the research team was also included in the introductory page of the questionnaire if further clarifications were required. On average, each respondent spent roughly 12 minutes to complete the full questionnaire.

### Data analysis

#### Construct validity and reliability.

Data analysis of the collected responses in this study was conducted using SPSS (version 29.00), while the demographic data of participants was summarised via descriptive statistics. The questionnaire’s validity and reliability were assessed using various assessment techniques. Item response theory (IRT) was used to validate the knowledge domain while Exploratory factor analysis (EFA) was used to validate the practice domain. Besides this, Cronbach’s alpha was used to calculate the internal consistency and assess reliability, which is the extent of conformity between items in the questionnaire with a minimum acceptable value of 0.7 [25]. The degree of agreement between the items increases with a greater Cronbach's alpha value.

### Item response theory (IRT)

To assess the knowledge section of the questionnaire, IRT, which is a valuable approach for understanding how participants’ responses to individual questions correlate to their proficiency level in the assessed knowledge domain, was employed [25]. Specifically, the one-parameter logistic (1PL) model, i.e., Rasch model was employed with ltm package version 1.2.0. Rasch model is a model used to evaluate participants’ performances and increase researcher’s construct instrument quality and accuracy in which compromises parameter a (discrimination) and parameter b (difficulty) [26]. For evaluation of psychometric properties of the domain, certain cutoff values have been established. Parameter a (discrimination) is fixed at a value of a = 1.0 for all items, implying that all items on the test are equally discriminating and the participants gathered for answering the questionnaire have similar level knowledge and traits. As for parameter b (difficulty), it spans between -3 and +3 [27]. These parameters and target range enable the model to faithfully depict the data and generate dependable estimates of the underlying constructs.

### Exploratory factor analysis (EFA)

EFA is used to aid in the identification of underlying inter-correlations among the variables of ordinal data (i.e., the Likert scale) and reduce the data to a smaller number of factors [28]. Varimax rotations are used to maximise each item's loading factor on the extracted respondents. Items that have a factor loading of minimum 0.3 are retained within the questionnaire while others will be removed to find items that meet acceptable construct validity standards [29].

## Results

### Sociodemographic characteristics of the participants

The study enrolled 52 participants with a mean age of 32 ± 6.06. Findings revealed that the predominant demographic comprised female pharmacists (63.46%) and holds a minimum qualification in Bachelor of Pharmacy degree (75%). Most of these professionals graduated from private universities (59.62%) and were mainly fully registered pharmacists (94.23%). These pharmacists have varying levels of working experience, with an average of 9 years. Notably, most of the participants were inpatient pharmacists (42.31%) as detailed in Table 3.

**Table 3:** Social demographic characteristics of participants (n = 52).

Variables	Mean (SD)	Frequency	Percentage (%)
**Age (years)**	32 (6.06)		
**Gender**			
Female		33	63.46%
Male		19	36.54%
**Education**			
Bachelor’s degree		39	75.0%
Master degree		12	23.08%
PhD		0	0%
Others		1	1.92%
**Place of Education**			
Government		21	40.38%
Private		31	59.62%
**Pharmacist Qualification**			
Provisionally registered		3	5.77%
Fully registered		49	94.23%
**Job Experience (years)**	9 (5.92)		
**Jobscope**			
Ward		11	21.15%
Outpatient		9	17.31%
Inpatient		22	42.31%
Others		10	19.23%

This diverse sample of pharmacists are able to provide a thorough insight of their knowledge and practice in AMS in the oncology care settings in view of their education, experience and job scope in the hospital. This ensures that the findings are relevant and applicable to a wide range of healthcare professionals who are working in this specific field.

### Item response theory (IRT)

The IRT analysis of the knowledge section conducted via the Rasch model using SPSS 29 program uncovered the psychometric properties of the domain, detailed in Table 4.

**Table 4:** Results of the IRT analysis in the knowledge section (n = 52), where discrimination (a) = 1.0.

Items	b	Standard error	x^2^	P value
K1: Antimicrobial stewardship (AMS) is associated with increased mortality rate, morbidity rate and healthcare cost.	-0.352	0.322	8.856	0.451
K2: AMS in oncology only involves pharmacists.	-0.901	0.339	13.994	0.123
K3: AMS helps to promote the appropriate use of antimicrobials and preserve them for the future.	1.708	0.403	34.669	<0.001
K4: AMS should only be implemented in tertiary hospitals.	-0.710	0.331	6.389	0.700
K5: When the source of an infection is unclear, there should be an antimicrobial “time-out” after 24 hours of empirical therapy to evaluate the need for ongoing antimicrobial treatment.	0.247	0.321	6.378	0.702
K6: AMS was only implemented to reduce abuse and misuse of antimicrobials.	-0.528	0.325	11.287	0.257
K7: AMS are able to reduce antimicrobial therapy duration only.	-1.213	0.357	5.004	0.834
K8: Decreasing the emergence, selection and spread of antimicrobial resistance (AMR) by optimising antimicrobial use is the primary goal of AMS.	2.684	0.548	28.165	<0.001
K9: Pharmacists serve as the first point of contact for viral respiratory tract infections where inappropriate use of antibiotics are commonly seen.	0.247	0.321	11.151	0.265
K10: Pharmacists are drug experts in which their knowledge can be used to rationalise antibiotic use and prevent the emergence of AMR.	2.027	0.440	10.717	0.296
K11: Only pharmacists with formal training in infectious disease can promote AMS.	-0.710	0.331	7.354	0.600
K12: Majority of ambulatory patients were prescribed broad-spectrum antibiotics unnecessarily.	0.695	0.333	10.661	0.300
K13: AMR only occurs as a result of self-medication.	-0.440	0.323	7.296	0.606
K14: Over usage of antimicrobials will lead not only to AMR but also fungal and virus resistance.	0.888	0.341	12.202	0.202
K15: Inappropriate usage of antimicrobials will not lead to an increasing rate of multidrug resistant organisms and disruption of healthy microbiomes.	-0.440	0.323	10.317	0.325
K16: Poor hand hygiene is the main contributing factor of AMR.	-0.010	0.319	5.908	0.749
**K17: Lack of proper identification of causative pathogens leads to the misuse of antimicrobials.**	**3.004**	0.619	7.117	0.625
K18: Cancer patients are at higher likelihood of developing antibiotic resistance through selection pressure due to needing multi-drug regimens to treat ongoing/multiple infections.	1.860	0.419	6.328	0.707
K19: Antimicrobial therapy prescribed in oncology patients is always considered appropriate and concordant with guidelines.	-1.001	0.344	25.382	0.003
K20: Use of treatment protocols to promote compliance with local and national guidelines are easy to effectively implement in cancer patients to improve appropriate antimicrobial prescribing.	1.094	0.353	16.791	0.052
K21: Clinical guidelines are developed to focus on the approach towards febrile neutropenia, antifungal prophylaxis in neutropenia as well as CMV treatment and prophylaxis.	1.570	0.389	24.558	0.004
K22: Clinical guidelines help to reduce prolonged antibiotic or antifungal therapy without impacting clinical outcome.	2.027	0.440	9.767	0.370
K23: Guidelines for clinical practices are recommended to be developed collaboratively by an infectious disease physician (ID) and a pharmacist with ID training to ensure comprehensive expertise.	1.860	0.419	7.457	0.590
K24: Cancer patients are less prone to infection and therefore timely administration of a right antimicrobial is not required.	-1.001	0.344	14.606	0.102
K25: Prolonged episodes of neutropenia and repeated courses of immunosuppressive agents do not diminish the susceptibility of cancer patients to infections.	-0.440	0.323	14.970	0.092
K26: Cancer patients have a lower mortality rate from a fatal infection than a person without cancer.	-1.325	0.365	9.193	0.420
K27: In patients who have developed antibiotic resistance, chemotherapy encourages microbe evolution and mutant bacteria emergence.	0.334	0.323	15.462	0.079
K28: Intravenous to oral de-escalation strategy is not a main consideration for infection control in oncology patients.	-1.000	0.344	15.475	0.079
K29: Infections can compromise patients’ treatment outcomes in the oncology care and delay chemotherapy treatment.	2.027	0.440	26.693	0.002
K30: Antibiotic usage will alter gut microbiome leading to dysbiosis which predispose patients to increased risk of infection including cancer.	0.790	0.337	12.052	0.210
K31: In patients with cancer, formulary management should include the evaluation of agents used in antibacterial prophylaxis during neutropenia associated with cytotoxic chemotherapy.	1.441	0.378	9.154	0.423
K32: Risk-stratification of patients should not be used when determining the treatment of neutropenic fever and prophylaxis indications in neutropenic patients with cancer.	-0.804	0.335	12.647	0.179
K33: Quinolone prophylaxis should be used in all neutropenic patients.	-1.001	0.344	14.716	0.099
K34: Cancer patients are more prone to opportunistic infections compared to non-cancer patients.	2.027	0.440	10.719	0.295
K35: Among antibiotics, decreased drug absorption, inhibition of renal excretion, and inhibition or induction of metabolism are common PK drug interactions.	1.860	0.419	26.113	0.002
K36: The effectiveness of the chemotherapeutics is not affected by resistance developed in the tumour tissue.	-0.901	0.339	11.461	0.245
K37: The combination of cancer chemotherapy and antibiotic use will promote antibiotic resistance mutations in cancer patients.	-0.010	0.319	11.954	0.216
K38: Chemotherapy contributes to the emergence of antibiotic-resistant bacteria within the gut while in combination with antibiotics, promotes pathogen overgrowth and translocation into the bloodstream.	0.511	0.327	9.229	0.416
K39: When used prophylactically, empirically, or therapeutically to manage infections, broad spectrum antibiotics have the potential to modify microbiomes, which in turn may change how cancer patients respond to treatment.	0.334	0.323	19.338	0.022
K40: Chemotherapy is likely to produce new antimicrobial resistance in the gut microbiota by deactivating the bacterial SOS system.	-0.010	0.319	8.595	0.475
K41: Antimicrobial drugs and anticancer drugs share common mechanisms of action and resistance.	-0.180	0.320	8.765	0.459
K42: There is no association between broad-spectrum antibiotics usage and reduced clinical response to immunotherapy in cancer patients.	-1.212	0.357	19.416	0.022
K43:Ciprofloxacin and vancomycin may cause delay in methotrexate elimination in cancer patients leading to severe toxicity.	0.247	0.321	9.196	0.419
K44: Cisplatin and aminoglycoside antibiotics have additive ototoxicity when used concurrently.	0.989	0.346	7.856	0.549
K45: Cisplatin may decrease the effectiveness of ciprofloxacin.	-0.901	0.339	7.637	0.571
K46: Concomitant usage of fluconazole and ondansetron may lead to QTc prolongation.	0.790	0.337	8.113	0.523
K47: The combination of Bactrim (trimethoprim+sulfamethoxazole) and methotrexate can lead to non-megaloblastic anaemia.	0.511	0.327	7.928	0.541
K48: Penicillins can reduce the hepatic excretion of methotrexate which leads to increased systemic exposure of methotrexate.	0.334	0.323	11.536	0.241
K49: Cancer patients who receive voriconazole will experience visual and auditory hallucinations due to elevated serum voriconazole concentrations.	0.161	0.320	14.787	0.097
K50: Itraconazole will interact with cyclophosphamide which is used in the management and treatment of neoplasms leading to altered cyclophosphamide metabolite levels and increased hepatotoxicity.	0.247	0.321	9.304	0.410

The sub- domains are AMS (K1, K2, K3, K4 and K5), goals of AMS (K6, K7 and K8), roles of pharmacist in AMS (K9, K10 and K11), trends in AMR (K12), risk factors of AMR occurrence (K13, K14, K15, K16, K17 and K18), clinical guidelines and protocols (K19, K20, K21, K22 and K23), infections in cancer patients (K24, K25, K26, K27, K28, K29 and K30), management of infection in cancer patients (K31, K32 and K33), PK-PD of antimicrobials in cancer patients (K34 and K35), association between chemotherapy and AMR (K36, K37, K38, K39, K40 and K41), association between immunotherapy and AMR (K42), drug-drug interactions between antimicrobial and chemotherapy (K43, K44, K45, K46, K47, K48, K49 and K50). The parameter b (difficulty) of items indicated that most were within the range of -3 to +3 except for item K17 which was removed from the questionnaire due to its’ surpassed value from the acceptance criteria. The remaining items were beyond the cut-off values.

According to the item-goodness-of-fit, only K3, K8, K19, K21, K29, K35, K39 and K42 (P < 0.05) fit well within the criteria while the rest exceed the significance level. However, in view of the item importance and relevance within the questionnaire, the remaining items are retained under the experts’ advice.

Furthermore, the average Cronbach alpha coefficient obtained across all of the knowledge items is 0.763 indicating that there is a high level of internal consistency among the items.

### Exploratory factor analysis (EFA)

For the practice domain, principal analysis in the SPSS program has suggested a 14-factor model extraction based on the scree plot and eigenvalue criterion (≥1). However, for better interpretability, the EFA was conducted with six factors instead, according to the domains that we have created initially. Via the rotation of factors with the Varimax method, all of the 56 items in the practice domain have an acceptable factor loading of > 0.3, indicating their relevance to the factors. Therefore, all of these items were retained in the questionnaire.

The six factors are assessment and decision making (items P1, P2, P3, P4, P5, P6 and P7), the implementation of AMS strategies (P8, P9, P10, P11, P12, P13, P14, P15, P16, P17, P18, P19, P20, P21, P22, P23, P24 and P25), interdisciplinary collaboration (P26, P27, P28, P29 and P30), patient education and counselling (P31, P32, P33, P34, P35 and P36), pharmacists’ education and training (P37, P38, P39, P40, P41, P42, P43, P44 and P45) and management of drug-drug interactions between chemotherapy and antimicrobials (P46, P47, P48, P49, P50, P51, P52, P53, P54, P55 and P56) as shown in Table 5.

**Table 5:** Results of the EFA of the practice domain.

Factors	Items	Factor loading	Cronbach’s alpha
**Assessment and Decision Making**	P1: I recommend the most appropriate antibiotic therapy for cancer patients based on local epidemiologic data, patient demographics and medication cost.	0.614	0.277
	P2: I recommend antibiotics to patients without considering institutional guidelines.	0.629	
	P3: I am confident to check guideline compliance of antimicrobial prescriptions.	0.405	
	P4: I dispense all repeat antimicrobial prescriptions without confirming its necessity.	0.610	
	P5: When establishing a patient-tailored empirical therapy plan, I take into account the patient's prior history of multidrug-resistant gram negative colonisation and institutional antibiograms or stratified antibiograms.	0.630	
	P6: When recommending AMS interventions to my consultants, I always provide evidence that comes from international guidelines or primary literature.	0.547	
	P7: I am aware of local antimicrobial resistance patterns.	0.493	
**Implementation of AMS Strategies**	P8: I review every prescription for appropriate antimicrobial use and intervene when necessary.	0.518	0.820
	P9: I frequently adjust antimicrobial doses based on patient-specific factors such as renal function, obesity or drug interactions.	0.645	
	P10: I always take a patient's antimicrobial allergies and adverse reactions into consideration	0.438	
	P11: I engage in de-escalation strategies by narrowing the spectrum of antimicrobial therapy before culture results are available in oncology patients.	0.526	
	P12: I recommend changing to a more suitable antibiotic when it is appropriate.	0.706	
	P13: I obtain blood cultures for patients with suspected infections.	0.528	
	P14: I recommend broad-spectrum antibiotics (e.g., amoxicillin + clavulanic acid) after blood culture results are available as first-line treatment options.	0.641	
	P15: I often involve myself in MedsChecks and/or Home Medicines Review (HMR) to ensure patients do not take antibiotics unnecessary.	0.749	
	P16: I review all restricted antimicrobials orders and make recommendations as appropriate.	0.518	
	P17: I am able to identify opportunities for AMS through the continuum of patient care in hospital pharmacies, communities pharmacies and/or transitions of care.	0.672	
	P18: I provide recommendations for de-escalation (e.g., stopping an unnecessary agent, de-escalating from broad-spectrum IV antibiotic to narrower-spectrum IV antibiotic or de-escalating from IV antibiotic to oral antibiotic) for patients that deem suitable after reviewing patient’s antibiotic orders.	0.525	
	P19: I monitor hospital antibiotic usage and antibiotic-resistant bacteria trends.	0.591	
	P20: I prescribe antibiotic prophylaxis to all cancer patients.	0.575	
	P21: I prescribe antibiotic prophylaxis to cancer patients with febrile neutropenia indefinitely.	0.578	
	P22: I conduct structured interviews, oral challenges or skin testings for the purpose of de-labelling patient’s self-reported penicillin allergies.	0.390	
	P23: I ask my patients regarding their previous use of antibiotics if any.	0.702	
	P24: I ensure that there are clear indications for antibiotic use when prescribing.	0.705	
	P25: I find it challenging to develop efficacious AMS programmes in community and/or hospital pharmacists due to lack of infectious disease (ID) pharmacists.	0.399	
**Interdisciplinary Collaboration**	P26: I provide regular feedback to other healthcare professionals (e.g., oncologist, physicians) regarding administration, dispensing and monitoring of antibiotics in oncology patients.	0.485	0.677
	P27: I work together with other healthcare professionals to promote infection prevention and AMS.	0.441	
	P28: I report results of stewardship activities to the authorities and hospital committee only during free time.	0.428	
	P29: I actively participate in multidisciplinary rounds to discuss antimicrobial therapy and stewardship strategies for oncology patients.	0.584	
	P30: I communicate with prescribers if I am unsure about the appropriateness of an antibiotic prescription.	0.628	
**Patient Education and Counselling**	P31: I educate oncology patients regarding the importance of antimicrobial adherence and potential side effects.	0.423	0.794
	P32: I always try to utilise best communication practices when counselling on antibiotics to ensure patient's take their medications properly.	0.847	
	P33: I always address any medication-related queries or concerns during my counselling of antibiotics to enhance adherence.	0.842	
	P34: I provide patients with information leaflets about their infections and prescribed antimicrobials during counselling.	0.436	
	P35: I recommend my patients to go to their GPs for enquiry regarding vaccine preventable infections.	0.349	
	P36: I make efforts to prevent or reduce transmission of infections within the community.	0.532	
**Pharmacists’ Education and Training**	P37: My tertiary education has prepared me to implement AMS in my current practice.	0.643	0.753
	P38: I participate in AMS programmes.	0.555	
	P39: I only attend training sessions when I’m free.	0.455	
	P40: I participate in a mentoring programme to offer insights and guidance to other healthcare professionals.	0.457	
	P41: I do not think pharmacists are required to be updated on the current practice guidelines for antimicrobial use.	0.656	
	P42: I am capable of demonstrating understanding, competence, skills and evidence-based knowledge in antimicrobial stewardship.	0.556	
	P43: I am able to define the terms such as AMS, empiric therapy, directed therapy and de-escalation as well as understand the concept behind them.	0.602	
	P44: I am confident in training other pharmacy interns and pharmacists on AMS.	0.756	
	P45: I promote optimal antimicrobial use by providing education, developing and implementing clinical practice guidelines.	0.596	
**Management of DDI between Chemotherapy and Antimicrobials**	P46: I make changes in drug administration timing, drug dosage and provide additional patient monitoring to manage potential DDI.	0.731	0.721
	P47: I do not take into account the potential for drug-drug interaction with chemotherapy when selecting appropriate antimicrobials (i.e., ciprofloxacin) for the patient.	0.705	
	P48: I prescribe amphotericin B regularly for patients taking cisplatin as it is safe to do so.	0.728	
	P49: I have sufficient knowledge about the most common interacting drugs used in cancer patients.	0.356	
	P50: I do not closely monitor for methotrexate toxicity when the patient is taking it concurrently with Bactrim (i.e., sulfamethoxazole/trimethoprim) as there is no DDI among the two.	0.444	
	P51: I do not conduct additional monitoring when ondansetron and fluconazole are used concurrently.	0.526	
	P52: I monitor patient’s creatinine clearance closely when they are on a combination of cyclosporine and aminoglycoside (e.g., gentamicin).	0.533	
	P53: I check databases such as drug interaction softwares to confirm the safety of all medications when screening the medication chart of the patient.	0.432	
	P54: I recommend therapeutic drug monitoring to optimise individual medication regimens when an inappropriate combination of drugs with interactions has to be continued.	0.414	
	P55: I am aware that drug-drug interactions between cisplatin and ciprofloxacin can cause nephrotoxic effects.	0.492	
	P56: I monitor the concentration level of antibiotics and antitumor drugs to avoid side reactions.	0.707	

The reliability of these questions on assessment and decision making, as well as interdisciplinary collaboration are 0.277 and 0.677 respectively, showing that the items have a low internal consistency within their domains. However, these subdomains are crucial in evaluating pharmacists’ roles in AMS within oncology care. Therefore, the removal of these subdomains will significantly reduce the questionnaire’s scope and relevance. It would be beneficial for future studies to conduct an item total correlation analysis to identify items that contribute to the low internal consistency. Nevertheless, the subdomains of the implementation of AMS strategies, patient education and counselling, pharmacists’ education and training, as well as management of drug-drug interactions between chemotherapy and antimicrobials have high internal consistencies of 0.820, 0.794, 0.753 and 0.721 respectively.

### Internal consistency reliability of the 105-item KP-AMS-OC-Q

As explained above, K17 was removed from the questionnaire due to its’ surpassed value from the acceptance criteria. This leads to a final 105-item KP-AMS-OC-Q in which its’ internal consistency was then assessed via Cronbach’s alpha within both knowledge and practice domains, as well as across each individual item.

The 105-item KP-AMS-OC-Q showed a notable overall Cronbach’s alpha value of 0.889, indicating a strong internal consistency reliability. In particular, the knowledge domain achieved a value of 0.763, while the practice domain achieved a high value of internal consistency with a Cronbach’s alpha of 0.921 as demonstrated in Table 6.

**Table 6:** Internal consistency reliability of the final 105-item KP-AMS-OC-Q (n = 52).

KP-AMS-OC-Q (n = 52)	Cronbach’s alpha
Knowledge domain	0.763
Practice domain	0.921
Overall	0.889

## Discussion

Over the years, existing AMS knowledge assessment tools for pharmacists and pharmacy students have provided useful context for evaluating the comprehensiveness and relevance of our instrument. For instance, a study by Mubarak et. al. in 2021 [30] assessed AMS-related education among pharmacy students, highlighting key areas of AMS knowledge that may also be relevant to practicing pharmacists. However, our study is the first study of its kind that aims to develop and validate a questionnaire focusing on the knowledge and practice of AMS in oncology care, filling a significant gap in existing research and assisting future evaluation of pharmacists’s AMS levels. The development process of the KP-AMS-OC-Q, involving rigorous psychometric analysis, has resulted in an instrument with satisfactory content validity, face validity, and reliability, making it a valuable tool for evaluating AMS practices among pharmacists in this specialized field.

This research serves as a pilot testing for content and construct validity and reliability of the questionnaire before disseminating it to the greater population of pharmacists. Knowledge and practice domains were assessed using psychometric instruments such as IRT and EFA respectively. The IRT analysis of the knowledge domain, conducted via the Rasch model, revealed that the majority of items demonstrated appropriate psychometric properties, with difficulty parameters within the acceptable range. The removal of item K17 due to its deviation from the recommended range reflects the stringent criteria employed to ensure the accuracy and validity of the questionnaire. The overall Cronbach's alpha coefficient of 0.763 for the knowledge domain indicates a high level of internal consistency, confirming that the items effectively measure the intended constructs related to AMS in oncology care.

The EFA of the practice domain revealed a six-factor model that aligns well with the predefined domains, providing a strong structural foundation for the questionnaire. The decision to reduce the number of factors from the initial 14-factor suggestion to 6 was guided by the need for better interpretability without compromising the questionnaire's comprehensiveness. The acceptable factor loadings (>0.3) [29] across all 56 items further validate the relevance of these items to the respective factors. The internal consistency reliability of the practice domain was notably high, particularly in the subdomains related to AMS strategies implementation, patient education and counselling, pharmacists’ education and training, and management of drug-drug interactions between chemotherapy and antimicrobials. However, the lower reliability observed in the subdomains of assessment and decision-making and interdisciplinary collaboration suggests areas that may benefit from further refinement or additional research.

The final 105-item KP-AMS-OC-Q, with an overall Cronbach’s alpha of 0.889, demonstrates strong internal consistency reliability, making it a reliable tool for assessing pharmacists’ knowledge and practices towards AMS in oncology care. The high reliability across both the knowledge (0.763) and practice (0.921) domains highlights the instrument's potential utility in both research and clinical practice. The removal of item K17, which did not meet the acceptance criteria, further ensures that the final instrument is both statistically robust and practically relevant.

In spite of the extensive nature of this questionnaire, the result of the pilot testing revealed that participants are able to fully complete it within an average timeframe of approximately 12 minutes. This, in addition to the favourable findings from the psychometric analysis, further support the questionnaire applicability and viability if conducted in a larger sample size.

### Limitations of the study

Notable limitations are present in our study. Firstly, a cross-sectional design does not allow the assessment of changes in pharmacists’ AMS knowledge and practices over time. Hence, future research could address this limitation by incorporating longitudinal follow-up studies to observe changes in knowledge and practices of pharmacists over an extended period. Secondly, a larger sample size of at least 100 participants would present a greater validation for future studies to conduct EFA [31]. A greater response rate can further increase the generalizability of the questionnaire’s results to the pharmacist population. Given that there are less hospitals in Malaysia which have oncology specialities, pharmacists who have responded to the questionnaire are primarily hospital pharmacists who may not be specialised in the setting, hence limiting the quantity of data obtained. Besides, future research could also consider using confirmatory factor analysis (CFA) for a better reliability and validity of the psychometric evaluation of KP-AMS-OC-Q. Furthermore, the nature of the questionnaire may introduce potential for social desirability bias that will lead to overestimation of pharmacist adherence to antimicrobial stewardship practices. Therefore, future studies may incorporate objective assessments such as direct observation to improve the transparency of the results.

## Conclusion

The Knowledge and Practice of Antimicrobial Stewardship in Oncology Care Questionnaire (KP-AMS-OC-Q) has been diligently developed and validated to specifically target hospital pharmacists in Malaysia as the primary study participants. Following a thorough psychometric evaluation, the final questionnaire comprises 112 items (7 items in sociodemographic information, 49 items in the knowledge domain and 56 items in the practice domain). This instrument plays a crucial role in healthcare settings, particularly in enhancing the effectiveness of AMS programs within oncology care and will help identify gaps requiring further education and training. Thus, this will lead to enhanced patient care, more efficient antimicrobial use and strengthened efforts to combat antimicrobial resistance in oncology patients.

## Supporting information

S1 File Supporting information “Questionnaire Tool”. (DOCX)

S2 File “Statitical Raw Data”. (ZIP)
