# Supplementary material for: Development and validation of a questionnaire assessing pharmacists' knowledge and practice towards antimicrobial stewardship in oncology care
Source: PLoS One. 2025 May 23;20(5):e0321551. doi: 10.1371/journal.pone.0321551 (PMC12101630; doi:10.1371/journal.pone.0321551)
Supplement: S1 File — (DOCX) [file pone.0321551.s001.docx]

**Development and Validation of a Questionnaire Assessing Pharmacists' Knowledge and Practice towards Antimicrobial Stewardship in Oncology Care**

***Section 1: Social demographics***

| Questions | Response |
| --- | --- |
| Age | ___ years old. |
| Gender | ( )Woman  ( )Man |
| Education | My qualification in pharmacy is __.  ( ) Bachelor’s degree  ( ) Master degree  ( ) PhD  ( ) Others: ____ (Please specify) |
| Place of Education | I graduated from a ___ university.  ( ) Government  ( ) Private |
| Pharmacist Qualification | I am a __ pharmacist.  ( ) Provisionally registered  ( ) Fully registered |
| Job Experience | I have been working as a pharmacist for __ years. |
| Jobscope | I’m currently working as a __ pharmacist.  ( ) Ward  ( ) Outpatient  ( ) Inpatient  ( ) Others: ___ (Please specify) |

***Section 2 Knowledge***

***The following 49 statements will test your knowledge towards antimicrobial stewardships in oncology care. Please select the most appropriate option.***

***Please select "Yes" if you think that the statement is right.***

***Please select "No" if you think that the statement is wrong.***

***Please select "Unsure" if you are not sure about the answer.***

| K1 | Antimicrobial stewardship (AMS) is associated with increased mortality rate, morbidity rate and healthcare cost.  ( ) Yes  ( ) No  ( ) Unsure |
| --- | --- |
| K2 | AMS in oncology only involves pharmacists.  ( ) Yes  ( ) No  ( ) Unsure |
| K3 | AMS helps to promote the appropriate use of antimicrobials and preserve them for the future.  ( ) Yes  ( ) No  ( ) Unsure |
| K4 | AMS should only be implemented in tertiary hospitals.  ( ) Yes  ( ) No  ( ) Unsure |
| K5 | When the source of an infection is unclear, there should be an antimicrobial “time-out” after 24 hours of empirical therapy to evaluate the need for ongoing antimicrobial treatment.  ( ) Yes  ( ) No  ( ) Unsure |
| K6 | AMS was only implemented to reduce abuse and misuse of antimicrobials.  ( ) Yes  ( ) No  ( ) Unsure |
| K7 | AMS are able to reduce antimicrobial therapy duration only.  ( ) Yes  ( ) No  ( ) Unsure |
| K8 | Decreasing the emergence, selection and spread of antimicrobial resistance (AMR) by optimising antimicrobial use is the primary goal of AMS.  ( ) Yes  ( ) No  ( ) Unsure |
| K9 | Pharmacists serve as the first point of contact for viral respiratory tract infections where inappropriate use of antibiotics are commonly seen.  ( ) Yes  ( ) No  ( ) Unsure |
| K10 | Pharmacists are drug experts in which their knowledge can be used to rationalise antibiotic use and prevent the emergence of AMR.  ( ) Yes  ( ) No  ( ) Unsure |
| K11 | Only pharmacists with formal training in infectious disease can promote AMS.  ( ) Yes  ( ) No  ( ) Unsure |
| K12 | Majority of ambulatory patients were prescribed broad-spectrum antibiotics unnecessarily.  ( ) Yes  ( ) No  ( ) Unsure |
| K13 | AMR only occurs as a result of self-medication.  ( ) Yes  ( ) No  ( ) Unsure |
| K14 | Over usage of antimicrobials will lead not only to AMR but also fungal and virus resistance.  ( ) Yes  ( ) No  ( ) Unsure |
| K15 | Inappropriate usage of antimicrobials will not lead to an increasing rate of multidrug resistant organisms and disruption of healthy microbiomes.  ( ) Yes  ( ) No  ( ) Unsure |
| K16 | Poor hand hygiene is the main contributing factor of AMR.  ( ) Yes  ( ) No  ( ) Unsure |
| ~~K17~~ | ~~Lack of proper identification of causative pathogens leads to the misuse of antimicrobials.~~  ~~( ) Yes~~  ~~( ) No~~  ~~( ) Unsure~~ |
| K18 | Cancer patients are at higher likelihood of developing antibiotic resistance through selection pressure due to needing multi-drug regimens to treat ongoing/multiple infections.  ( ) Yes  ( ) No  ( ) Unsure |
| K19 | Antimicrobial therapy prescribed in oncology patients is always considered appropriate and concordant with guidelines.  ( ) Yes  ( ) No  ( ) Unsure |
| K20 | Use of treatment protocols to promote compliance with local and national guidelines are easy to effectively implement in cancer patients to improve appropriate antimicrobial prescribing.  ( ) Yes  ( ) No  ( ) Unsure |
| K21 | Clinical guidelines are developed to focus on the approach towards febrile neutropenia, antifungal prophylaxis in neutropenia as well as CMV treatment and prophylaxis.  ( ) Yes  ( ) No  ( ) Unsure |
| K22 | Clinical guidelines help to reduce prolonged antibiotic or antifungal therapy without impacting clinical outcome.  ( ) Yes  ( ) No  ( ) Unsure |
| K23 | Guidelines for clinical practices are recommended to be developed collaboratively by an infectious disease physician (ID) and a pharmacist with ID training to ensure comprehensive expertise.  ( ) Yes  ( ) No  ( ) Unsure |
| K24 | Cancer patients are less prone to infection and therefore timely administration of a right antimicrobial is not required.  ( ) Yes  ( ) No  ( ) Unsure |
| K25 | Prolonged episodes of neutropenia and repeated courses of immunosuppressive agents do not diminish the susceptibility of cancer patients to infections.  ( ) Yes  ( ) No  ( ) Unsure |
| K26 | Cancer patients have a lower mortality rate from a fatal infection than a person without cancer.  ( ) Yes  ( ) No  ( ) Unsure |
| K27 | In patients who have developed antibiotic resistance, chemotherapy encourages microbe evolution and mutant bacteria emergence.  ( ) Yes  ( ) No  ( ) Unsure |
| K28 | Intravenous to oral de-escalation strategy is not a main consideration for infection control in oncology patients.  ( ) Yes  ( ) No  ( ) Unsure |
| K29 | Infections can compromise patients’ treatment outcomes in the oncology care and delay chemotherapy treatment.  ( ) Yes  ( ) No  ( ) Unsure |
| K30 | Antibiotic usage will alter gut microbiome leading to dysbiosis which predispose patients to increased risk of infection including cancer.  ( ) Yes  ( ) No  ( ) Unsure |
| K31 | In patients with cancer, formulary management should include the evaluation of agents used in antibacterial prophylaxis during neutropenia associated with cytotoxic chemotherapy.  ( ) Yes  ( ) No  ( ) Unsure |
| K32 | Risk-stratification of patients should not be used when determining the treatment of neutropenic fever and prophylaxis indications in neutropenic patients with cancer.  ( ) Yes  ( ) No  ( ) Unsure |
| K33 | Quinolone prophylaxis should be used in all neutropenic patients.  ( ) Yes  ( ) No  ( ) Unsure |
| K34 | Cancer patients are more prone to opportunistic infections compared to non-cancer patients.  ( ) Yes  ( ) No  ( ) Unsure |
| K35 | Among antibiotics, decreased drug absorption, inhibition of renal excretion, and inhibition or induction of metabolism are common PK drug interactions.  ( ) Yes  ( ) No  ( ) Unsure |
| K36 | The effectiveness of the chemotherapeutics is not affected by resistance developed in the tumour tissue.  ( ) Yes  ( ) No  ( ) Unsure |
| K37 | The combination of cancer chemotherapy and antibiotic use will promote antibiotic resistance mutations in cancer patients.  ( ) Yes  ( ) No  ( ) Unsure |
| K38 | Chemotherapy contributes to the emergence of antibiotic-resistant bacteria within the gut while in combination with antibiotics, promotes pathogen overgrowth and translocation into the bloodstream.  ( ) Yes  ( ) No  ( ) Unsure |
| K39 | When used prophylactically, empirically, or therapeutically to manage infections, broad spectrum antibiotics have the potential to modify microbiomes, which in turn may change how cancer patients respond to treatment.  ( ) Yes  ( ) No  ( ) Unsure |
| K40 | Chemotherapy is likely to produce new antimicrobial resistance in the gut microbiota by deactivating the bacterial SOS system.  ( ) Yes  ( ) No  ( ) Unsure |
| K41 | Antimicrobial drugs and anticancer drugs share common mechanisms of action and resistance.  ( ) Yes  ( ) No  ( ) Unsure |
| K42 | There is no association between broad-spectrum antibiotics usage and reduced clinical response to immunotherapy in cancer patients.  ( ) Yes  ( ) No  ( ) Unsure |
| K43 | Ciprofloxacin and vancomycin may cause delay in methotrexate elimination in cancer patients leading to severe toxicity.  ( ) Yes  ( ) No  ( ) Unsure |
| K44 | Cisplatin and aminoglycoside antibiotics have additive ototoxicity when used concurrently.  ( ) Yes  ( ) No  ( ) Unsure |
| K45 | Cisplatin may decrease the effectiveness of ciprofloxacin.  ( ) Yes  ( ) No  ( ) Unsure |
| K46 | Concomitant usage of fluconazole and ondansetron may lead to QTc prolongation.  ( ) Yes  ( ) No  ( ) Unsure |
| K47 | The combination of Bactrim (trimethoprim+sulfamethoxazole) and methotrexate can lead to non-megaloblastic anaemia.  ( ) Yes  ( ) No  ( ) Unsure |
| K48 | Penicillins can reduce the hepatic excretion of methotrexate which leads to increased systemic exposure of methotrexate.  ( ) Yes  ( ) No  ( ) Unsure |
| K49 | Cancer patients who receive voriconazole will experience visual and auditory hallucinations due to elevated serum voriconazole concentrations.  ( ) Yes  ( ) No  ( ) Unsure |
| K50 | Itraconazole will interact with cyclophosphamide which is used in the management and treatment of neoplasms leading to altered cyclophosphamide metabolite levels and increased hepatotoxicity.  ( ) Yes  ( ) No  ( ) Unsure |

***Section 3 Practice***

***The following 56 statements will assess your practice towards antimicrobial stewardship in oncology care.***

***Please choose the most appropriate response for you in each statement, namely "Never", "Rarely", "Sometimes", "Often" or "Always".***

|  | Questions | Never | Rarely | Sometimes | Often | Always |
| --- | --- | --- | --- | --- | --- | --- |
| P1 | I recommend the most appropriate antibiotic therapy for cancer patients based on local epidemiologic data, patient demographics and medication cost. |  |  |  |  |  |
| P2 | I recommend antibiotics to patients without considering institutional guidelines. |  |  |  |  |  |
| P3 | I am confident to check guideline compliance of antimicrobial prescriptions. |  |  |  |  |  |
| P4 | I dispense all repeat antimicrobial prescriptions without confirming its necessity. |  |  |  |  |  |
| P5 | When establishing a patient-tailored empirical therapy plan, I take into account the patient's prior history of multidrug-resistant gram negative colonisation and institutional antibiograms or stratified antibiograms. |  |  |  |  |  |
| P6 | When recommending AMS interventions to my consultants, I always provide evidence that comes from international guidelines or primary literature. |  |  |  |  |  |
| P7 | I am aware of local antimicrobial resistance patterns. |  |  |  |  |  |
| P8 | I review every prescription for appropriate antimicrobial use and intervene when necessary. |  |  |  |  |  |
| P9 | I frequently adjust antimicrobial doses based on patient-specific factors such as renal function, obesity or drug interactions. |  |  |  |  |  |
| P10 | I always take a patient's antimicrobial allergies and adverse reactions into consideration |  |  |  |  |  |
| P11 | I engage in de-escalation strategies by narrowing the spectrum of antimicrobial therapy before culture results are available in oncology patients. |  |  |  |  |  |
| P12 | I recommend changing to a more suitable antibiotic when it is appropriate. |  |  |  |  |  |
| P13 | I obtain blood cultures for patients with suspected infections. |  |  |  |  |  |
| P14 | I recommend broad-spectrum antibiotics (e.g. amoxicillin + clavulanic acid) after blood culture results are available as first-line treatment options. |  |  |  |  |  |
| P15 | I often involve myself in MedsChecks and/or Home Medicines Review (HMR) to ensure patients do not take antibiotics unnecessary. |  |  |  |  |  |
| P16 | I review all restricted antimicrobials orders and make recommendations as appropriate. |  |  |  |  |  |
| P17 | I am able to identify opportunities for AMS through the continuum of patient care in hospital pharmacies, communities pharmacies and/or transitions of care. |  |  |  |  |  |
| P18 | I provide recommendations for de-escalation (e.g. stopping an unnecessary agent, de-escalating from broad-spectrum IV antibiotic to narrower-spectrum IV antibiotic or de-escalating from IV antibiotic to oral antibiotic) for patients that deem suitable after reviewing patient’s antibiotic orders. |  |  |  |  |  |
| P19 | I monitor hospital antibiotic usage and antibiotic-resistant bacteria trends. |  |  |  |  |  |
| P20 | I prescribe antibiotic prophylaxis to all cancer patients. |  |  |  |  |  |
| P21 | I prescribe antibiotic prophylaxis to cancer patients with febrile neutropenia indefinitely. |  |  |  |  |  |
| P22 | I conduct structured interviews, oral challenges or skin testings for the purpose of de-labelling patient’s self-reported penicillin allergies. |  |  |  |  |  |
| P23 | I ask my patients regarding their previous use of antibiotics if any. |  |  |  |  |  |
| P24 | I ensure that there are clear indications for antibiotic use when prescribing. |  |  |  |  |  |
| P25 | I find it challenging to develop efficacious AMS programmes in community and/or hospital pharmacists due to lack of infectious disease (ID) pharmacists. |  |  |  |  |  |
| P26 | I provide regular feedback to other healthcare professionals (e.g. oncologist, physicians) regarding administration, dispensing and monitoring of antibiotics in oncology patients. |  |  |  |  |  |
| P27 | I work together with other healthcare professionals to promote infection prevention and AMS. |  |  |  |  |  |
| P28 | I report results of stewardship activities to the authorities and hospital committee only during free time. |  |  |  |  |  |
| P29 | I actively participate in multidisciplinary rounds to discuss antimicrobial therapy and stewardship strategies for oncology patients. |  |  |  |  |  |
| P30 | I communicate with prescribers if I am unsure about the appropriateness of an antibiotic prescription. |  |  |  |  |  |
| P31 | I educate oncology patients regarding the importance of antimicrobial adherence and potential side effects. |  |  |  |  |  |
| P32 | I always try to utilise best communication practices when counselling on antibiotics to ensure patient's take their medications properly. |  |  |  |  |  |
| P33 | I always address any medication-related queries or concerns during my counselling of antibiotics to enhance adherence. |  |  |  |  |  |
| P34 | I provide patients with information leaflets about their infections and prescribed antimicrobials during counselling. |  |  |  |  |  |
| P35 | I recommend my patients to go to their GPs for enquiry regarding vaccine preventable infections. |  |  |  |  |  |
| P36 | I make efforts to prevent or reduce transmission of infections within the community. |  |  |  |  |  |
| P37 | My tertiary education has prepared me to implement AMS in my current practice. |  |  |  |  |  |
| P38 | I participate in AMS programmes. |  |  |  |  |  |
| P39 | I only attend training sessions when I’m free. |  |  |  |  |  |
| P40 | I participate in a mentoring programme to offer insights and guidance to other healthcare professionals. |  |  |  |  |  |
| P41 | I do not think pharmacists are required to be updated on the current practice guidelines for antimicrobial use. |  |  |  |  |  |
| P42 | I am capable of demonstrating understanding, competence, skills and evidence-based knowledge in antimicrobial stewardship. |  |  |  |  |  |
| P43 | I am able to define the terms such as AMS, empiric therapy, directed therapy and de-escalation as well as understand the concept behind them. |  |  |  |  |  |
| P44 | I am confident in training other pharmacy interns and pharmacists on AMS. |  |  |  |  |  |
| P45 | I promote optimal antimicrobial use by providing education, developing and implementing clinical practice guidelines. |  |  |  |  |  |
| P46 | I make changes in drug administration timing, drug dosage and provide additional patient monitoring to manage potential DDI. |  |  |  |  |  |
| P47 | I do not take into account the potential for drug-drug interaction with chemotherapy when selecting appropriate antimicrobials (i.e. ciprofloxacin) for the patient. |  |  |  |  |  |
| P48 | I prescribe amphotericin B regularly for patients taking cisplatin as it is safe to do so. |  |  |  |  |  |
| P49 | I have sufficient knowledge about the most common interacting drugs used in cancer patients. |  |  |  |  |  |
| P50 | I do not closely monitor for methotrexate toxicity when the patient is taking it concurrently with Bactrim (i.e. sulfamethoxazole/trimethoprim) as there is no DDI among the two. |  |  |  |  |  |
| P51 | I do not conduct additional monitoring when ondansetron and fluconazole are used concurrently. |  |  |  |  |  |
| P52 | I monitor patient’s creatinine clearance closely when they are on a combination of cyclosporine and aminoglycoside (e.g. gentamicin). |  |  |  |  |  |
| P53 | I check databases such as drug interaction softwares to confirm the safety of all medications when screening the medication chart of the patient. |  |  |  |  |  |
| P54 | I recommend therapeutic drug monitoring to optimise individual medication regimens when an inappropriate combination of drugs with interactions has to be continued. |  |  |  |  |  |
| P55 | I am aware that drug-drug interactions between cisplatin and ciprofloxacin can cause nephrotoxic effects. |  |  |  |  |  |
| P56 | I monitor the concentration level of antibiotics and antitumor drugs to avoid side reactions. |  |  |  |  |  |
