# Supplementary material for: Development and validation of a questionnaire assessing pharmacists' knowledge and practice towards antimicrobial stewardship in oncology care
Source: PLoS One. 2025 May 23;20(5):e0321551. doi: 10.1371/journal.pone.0321551 (PMC12101630; doi:10.1371/journal.pone.0321551)
Supplement: S2 File — (ZIP) [file pone.0321551.s002.zip › print_release.pdf]

This is to certify that these images are supplied copyright-free by Colorfoto Ltd for reproduction, distribution and publication.

Photographer: Colorfoto
Customer: Fatima Blebil
Album(s): 7HE
Order key: 2063 8154 604485
Date of order: 17/10/2023

Images included in this print release:

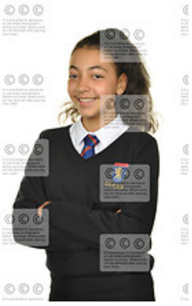	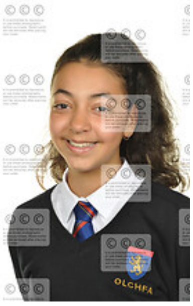	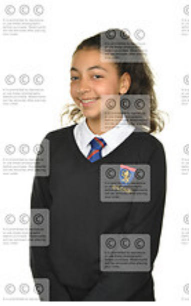
RJ19-18840.jpg	RJ19-18841.jpg	RJ19-18839.jpg
